# Integrated delivery of family planning and childhood immunisation services: a mixed methods assessment of service responsiveness

**DOI:** 10.1186/s12913-022-07983-7

**Published:** 2022-04-28

**Authors:** Jessie K. Hamon, Misozi Kambanje, Shannon Pryor, Alice S. Kaponda, Erick Mwale, Helen E. D. Burchett, Susannah H. Mayhew, Jayne Webster

**Affiliations:** 1 Department of Disease Control, London School of Hygiene & Tropical Medicine (LSHTM), Keppel Street, London, WC1E 7HT UK; 2 Save the Children, Blantyre, Malawi; 3 Save the Children, Washington D.C., USA; 4 Department of Public Health, Environments and Society, LSHTM, London, UK; 5 Department of Global Health and Development, LSHTM, London, UK

**Keywords:** family planning, childhood immunisations, integration, service delivery, responsiveness

## Abstract

**Background:**

Postpartum women represent a considerable share of the global unmet need for modern contraceptives. Evidence suggests that the integration of family planning (FP) with childhood immunisation services could help reduce this unmet need by providing repeat opportunities for timely contact with FP services. However, little is known about the clients’ experiences of FP services that are integrated with childhood immunisations, despite being crucial to contraceptive uptake and repeat service utilisation.

**Methods:**

The responsiveness of FP services that were integrated with childhood immunisations in Malawi was assessed using cross-sectional convergent mixed methods. Exit interviews with clients (*n*=146) and audits (*n*=15) were conducted in routine outreach clinics. Responsiveness scores across eight domains were determined according to the proportion of clients who rated each domain positively. Text summary analyses of qualitative data from cognitive interviewing probes were also conducted to explain responsiveness scores. Additionally, Spearman rank correlation and Pearson’s chi-squared test were used to identify correlations between domain ratings and to examine associations between domain ratings and client, service and clinic characteristics.

**Results:**

Responsiveness scores varied across domains: dignity (97.9%); service continuity (90.9%); communication (88.7%); ease of access (77.2%); counselling (66.4%); confidentiality (62.0%); environment (53.9%) and choice of provider (28.4%). Despite some low performing domains, 98.6% of clients said they would recommend the clinic to a friend or family member interested in FP. The choice of provider, communication, confidentiality and counselling ratings were positively associated with clients’ exclusive use of one clinic for FP services. Also, the organisation of services in the clinics and the providers’ individual behaviours were found to be critical to service responsiveness.

**Conclusions:**

This study establishes that in routine outreach clinics, FP services can be responsive when integrated with childhood immunisations, particularly in terms of the dignity and service continuity afforded to clients, though less so in terms of the choice of provider, environment, and confidentiality experienced. Additionally, it demonstrates the value of combining cognitive interviewing techniques with Likert questions to assess service responsiveness.

## Background

Approximately 218 million women have an unmet need for modern contraceptives in low- and middle-income countries [1], among which postpartum women make up a considerable share [2, 3]. Evidence suggests that the integration of family planning (FP) services with childhood immunisations could help reduce the unmet need among postpartum women by providing repeat opportunities for timely contact with FP services [4]. In some settings, studies have demonstrated that the integration of FP services with childhood immunisations is acceptable to clients and providers, and that it can result in increased contraceptive uptake with little to no negative impact on immunisations [5–10]. However, research on the integration of these two services has primarily focused on reproductive and behavioural outcomes and less is known about the client experience. This represents an important gap given the established link between the experiential quality of services and both contraceptive uptake and repeat use of health services [11–16].

A better understanding of the responsiveness of FP services that are integrated with childhood immunisations could help inform the design and implementation of high quality integrated FP services that are client-centred and rights-based [12, 17]. Service responsiveness is concerned with whether the experience of an individual’s interaction with a specific health service fulfils a set of ‘legitimate’ expectations or universally accepted ethical principles and non-clinical service standards [18–22]. In a review of factors determining the quality of FP services, Tessema *et al*. identified several studies investigating the quality of FP services that note the importance of responsiveness [23]. Despite this recognition, only two studies have directly investigated the responsiveness of FP services to date. First, a study in Niger demonstrated that low-cost interventions that improve service responsiveness can increase FP uptake when these services are integrated with curative and under-fives consultations [11]. Second, researchers in Sri Lanka developed an instrument to measure the responsiveness of FP services, assessed their responsiveness, and identified its correlates and determinants [24–26]. According to their research, the domains of responsiveness that are most relevant to FP services are the dignity, environment, choice, communication, confidentiality and ease of access afforded to clients [26].

In 2019, a case study of the responsiveness of FP services that were integrated with childhood immunisations in routine outreach clinics was conducted in Malawi. This case study documented clients’ and providers’ perspectives using mixed methods. The findings presented here comprise the first part of this study, which aimed to assess clients’ experiences of FP services that were integrated with childlhood immunisations and determine the factors associated with perceived responsiveness.

## Methods

The case study was conducted within a multi-faceted process evaluation carried out in Malawi, Benin, Kenya and Uganda, which interrogated the pathways to outcomes of an NGO-led intervention integrating the delivery of FP services with childhood immunisations in rural areas. In Malawi, the case study took place between June and July 2019 in routine public outreach clinics where the delivery of FP services was integrated into the existing Expanded Programme on Immunisation (EPI), which included childhood immunisations and growth monitoring services.

A cross-sectional convergent mixed methods design was used through which quantitative and qualitative results were combined to generate a comprehensive understanding of clients’ experiences [27]. The selection of this approach was informed by findings from a critical assessment of the WHO’s health systems responsiveness tool, which highlighted the importance of using mixed methods in the assessment of clients’ experiences of outpatient services [28]. Empirical data were collected through clinic audits and exit interviews with clients. Programme monitoring data were also consulted to identify the clinics’ FP client load on the day of the interviews. The methods and results from this study are reported here according to the GRAMMS guidelines for mixed methods studies in health services research [29].

### Study setting

A detailed account of the intervention that included the integrated delivery of FP services and childhood immunisations in routine outreach clinics is presented elsewhere [30]. In brief, the intervention was carried out between January 2015 and October 2019 in the Blantyre, Thyolo and Mwanza districts of Malawi. In these districts, childhood immunisation coverage was relatively high, and the unmet need for FP among married women was around 19.0% [31]. Broadly, the intervention intended to: 1) strengthen the capacity of providers to deliver quality integrated FP, childhood immunisation, and growth monitoring services in routine outreach clinics; 2) increase the retention of clients and reduce immunisation defaulters; 3) improve the availability of FP and immunisation supplies in clinics; and 4) improve community engagement around FP and immunisation service utilisation.

The outreach clinics included in the intervention were carried out each month during a single day in existing community buildings or in open spaces (e.g., under a tree). In these clinics, the organisation of services followed a defined client flow, which involved: a group health talk during which information about child development, FP, and immunisations was presented; the screening of clients for immunisation and FP services; the provision of growth monitoring and immunisations for children under five years of age; and the delivery of FP counselling and contraceptives [30]. This client flow was designed to streamline service delivery and was based on the assumption that clinics would be supported by a team of community volunteers and staffed by a minimum of four health surveillance assistants (HSAs). In Malawi, HSAs are paid community health workers attached to health centres, and are tasked with a wide range of health promotion responsibilities, including community-based delivery of FP services [32].

### Empirical data collection

#### Selection of study sites

At the time of data collection, FP services were integrated with childhood immunisations in 91 routine outreach clinics. Due to logistical and time constraints, clinics in Mwanza were excluded from the case study. Only clinics where FP services were integrated with childhood immunisations for 12 months or more prior to the start of data collection were considered for inclusion in the study based on the assumption that assessing the responsiveness in clinics where providers had delivered integrated services for at least a year would generate better insights. Of the 16 clinics that met this inclusion criterion, one was excluded due to a conflict in the data collection schedule. The study was ultimately carried out in 15 clinics across Blantyre (*n*=7) and Thyolo (*n*=8) districts, with data collected in each clinic during a single day.

#### Exit interviews with clients

Exit interviews were conducted in all 15 clinics by a team of nine experienced interviewers using a structured questionnaire. All eligible clients were recruited upon exit from the clinic based on the availability of interviewers until the sample size needs for the wider process evaluation were met. This sample size was based on an assumed percentage use of modern contraceptive methods of 50%, relative error 0.2, and design effect 3.0. Assuming 95% confidence and 80% power, a total of 192 exit interviews were required, with 13 per clinic as a target. Clients included in the responsiveness case study were those who sought both childhood immunisation and FP services on the day of the interview and were 18 years or older.

The structured questionnaire used to carry out interviews was employed in Chichewa and featured questions that were relevant to both this case study and to the wider process evaluation in which it was nested. Responsiveness-related questions focused on eight structural and behavioural domains (Table 1). Clients were asked to rate their experience of these domains using a five-point Likert scale, with responses ranging from ‘very good’ to ‘very bad’. They were also asked elaborative and hypothetical questions based on cognitive interviewing techniques to explain their ratings and to verify their interpretation of the Likert scale [33, 34]. That is, after each Likert question, the following probes were used: ‘can you explain what made you feel this way?’ and/or ‘what would it have taken for you to answer inversely?’. Responses to these questions were noted in English by the interviewers on the questionnaires. Additionally, clients were asked to rank the eight domains from most to least important and to confirm whether or not they had experienced key elements of FP counselling.

**Table 1:** Responsiveness domains and related questions included in the exit interviews

**Structural domains**	**Questions**
Environment	How was the cleanliness and space in the clinic?
Service continuity	How clear was the information about where/when to seek follow-up FP services?
Choice of provider	How was the freedom you had to choose a provider to assist you with FP in the clinic?
Ease of access	How easy was it for you to access this clinic today?
**Behavioural domains**	**Questions**
Dignity	How was the respect you received from the provider?
Confidentiality	How was the confidentiality provided to you by the FP provider?
Communication	How clear was the information you received from the provider?
Counselling	How was the attention the provider paid to your reproductive preferences?

The questionnaire was reviewed in-depth by the team of interviewers and piloted in two clinics to address language and logistical issues. The interviewers’ review revealed that ‘confidentiality’ would likely be interpreted by clients as whether the information they shared with providers was kept private. For this reason, a Chichewa word for ‘privacy’ was used, which conveyed confidentiality more broadly in terms of the privacy of information shared and the possibility of interacting with providers without others catching sight or overhearing.

#### Clinic audits

A structured questionnaire was used to perform audits of all 15 clinics on the day of the exit interviews, which documented the clinics’ resources and characteristics. Questions focused on the clinics’ infrastructure, number and cadre of providers, and stocks of FP and immunisation commodities. This questionnaire was piloted alongside the exit interview questionnaire in two clinics.

### Data management and analysis

Quantitative data from exit interviews and clinic audits were recorded on paper forms, double entered into EpiData, and exported into STATA 16 for analysis. Descriptive statistics were produced to summarise key clinic, service, and client characteristics. Domain-specific responsiveness scores were then determined using a two-step process. First, clients’ responses to the Likert questions were categorised into ‘positive’ and ‘negative’ ratings, with the middle point (moderate) of the scale added to the negative ratings. The decision to include the middle response among negative ratings was informed by the data from the cognitive interviewing probes, which revealed that moderate ratings predominantly represented negative experiences. Second, responsiveness scores were calculated as the proportion of clients who rated each domain positively (i.e. ‘good’ or ‘very good’). Spearman rank correlation was used to analyse the extent to which responsiveness ratings were correlated and Pearson’s chi-squared test was used to assess the association between responsiveness ratings and clinic-, service- and client-level factors. Additionally, for each respondent, pairwise comparisons of rankings across all domain pairs were carried out and then aggregated across individuals to generate an overall ranking of the relative importance of domains [35].

Qualitative data from the interviewers’ notes on the clients’ responses to the cognitive interviewing probes were imported into NVivo 12 for analysis. The aim of this analysis was primarily to examine the clients’ understanding of the Likert questions and scales; however further analyses were also performed using an inductive text summary approach to identify dominant themes among the clients’ responses [36]. These themes were then compared to the domain ratings from the exit interviews to explain the clients’ ratings and gain a better understanding of clients’ experiences.

## Results

### Client and clinic characteristics

A total of 146 exit interviews with clients were included in the case study. In all, 36.3% (n=53) were carried out in Blantyre district and 63.7% (n=93) were conducted in Thyolo district.

Of the clients who took part in the exit interviews, 53.1% were 18-24 years old, 91.8% had completed at least a primary education, and 93.8% were married. All were mothers to at least one child, 27.6% had two children or more under the age of five, 64.1% were repeat contraceptive users (i.e., collecting their usual method at the clinic), 95.6% reported having a husband who supported FP, 70.4% lived less than 45 minutes away from the clinic, 34.0% reported that the clinic where they were interviewed was the only one they used for FP services, and 98.6% said they would recommend the clinic to a friend or family member interested in FP.

**Fig. 1:**
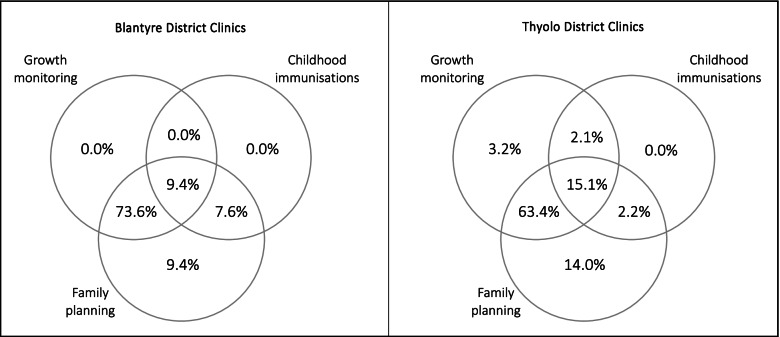
Reason for visiting the clinic on the day of the interview (*N*=146)

Additionally, only a small proportion of clients interviewed reported visiting the clinic with the intention to seek both immunisation and FP services, yet 73.6% of clients in Blantyre and 63.4% in Thyolo had intended to seek both growth monitoring and FP services on the day of the interview (Figure 1).

Overall, services were delivered in sites that lacked appropriate infrastructure and where a considerable number of clients sought services at the same time, which rendered the provision of services a challenge. That is, although 87.0% of respondents attended a clinic that met the staffing standards (four HSAs or more), only 60.3% attended a clinic that had a shelter and 31.0% were served in clinics that had more than one room available for the provision of services. Also, 50.0% sought services in a clinic where FP and childhood immunisations were delivered in the same space and 51.4% were served in a clinic that had a FP client load under 30 (range 12-61) on the day of the interview.

Despite NGO-led initiatives (e.g., training and routine supervision of HSAs) to strengthen the quality of FP counselling in the studied clinics, not all clients interviewed experienced comprehensive FP counselling. As summarised in Table 2, most clients reported only experiencing some elements of FP counselling.

**Table 2:** Elements of FP counselling reportedly experienced

Elements of FP counselling	Percent of sampled clients (***N***=146)
Heard the group health talk that contained information about FP	64.1%
Was asked how many more children are desired	19.9%
Was asked about past use of contraceptives	30.5%
Was asked about problems with past contraceptive use	43.3%
Was told how different contraceptive methods work	57.5%
Was told about possible contraceptive side effects	53.2%
Was told when to seek follow-up services	86.5%
Was told where to seek follow-up services	82.3%

### Importance of responsiveness domains

The respondents’ rankings of the eight responsiveness domains’ importance revealed that clients considered the clinic’s environment to be the most important domain. This was followed by confidentiality, service continuity, ease of access, dignity, choice of provider, counselling, and lastly, communication.

### Responsiveness scores

Responsiveness scores varied across domains (Table 3), with dignity (97.9%) rated most positively and the choice of provider (28.4%) rated least positively by clients. Overall, little variation was found between districts, and results from the Spearman rank correlation revealed no strong correlations between domain ratings.

**Table 3:** Responsiveness scores

Domains	Blantyre scores	Thyolo scores	Combined scores^a^
Dignity	96.2%	98.9%	97.9% (*N*=144)
Service continuity	92.3%	90.0%	90.9% (*N*=142)
Communication	88.5%	88.8%	88.7% (*N*=141)
Ease of access	73.6%	79.3%	77.2% (*N*=145)
Counselling	61.2%	69.4%	66.4% (*N*=134)
Confidentiality	58.5%	64.0%	62.0% (*N*=142)
Environment	53.9%	53.9%	53.9% (*N*=141)
Choice of provider	25.0%	30.3%	28.4% (*N*=141)

^a^*N* varied from 146 due to missing data

### Qualitative explanation of responsiveness scores

The analysis of interviewer notes from the cognitive interviewing probes confirmed that the Likert questions and scales were well understood by all respondents. That is, alignment was found between the intent of the questions and the clients’ responses, and the positive/negative nature of the ratings given by clients matched the explanations they provided for these. Additionally, the text summary analysis of this data helped explain the responsiveness scores, whilst revealing important factors influencing the clients’ perceptions. These findings are reported here for each domain.

#### Dignity

Among the many clients who rated dignity positively, several linked their rating to receiving the services or the help they felt they needed. Some specified that services were quick, whilst others said clients were not denied services, even when arriving late. Two clients also said they would have rated dignity less favourably if contraceptives were not available in the clinic. However, this domain’s positive ratings were most commonly explained by examples pertaining to the providers’ behaviour. Examples included providers behaving professionally, being kind, greeting clients, not shouting at clients, administering injections gently, and having a positive attitude.

#### Service continuity

Most clients who rated the service continuity positively said the date of their next visit was either verbally communicated to them or written in their health passport. Among these clients, almost all who reported having the date of their next visit recorded in their health passport rated this domain as 'very good'. Conversely, negative ratings were mainly linked to either not receiving any information about follow-up visits or being told to return within a given period (e.g. after three months) without a specified date.

#### Communication

Clients who rated the communication positively generally felt the information was clear and the advice provided was practical and helpful. They also said providers covered a wide range of topics, including: the benefits of FP, contraceptive options, and the management of side effects from contraceptives. Examples of providers creating a conducive environment for information sharing were also used to explain positive ratings. Such examples included providers 1) speaking loudly to ensure clients could hear the group health talk, 2) creating a friendly environment, and 3) encouraging clients to ask questions. Among the clients who rated this domain negatively, most reported not hearing the heath talk (e.g., because they reached the clinic late) or not receiving any FP counselling, and several said the information they received was incomplete or lacking details.

#### Ease of access

Many of the clients who rated their ease of access positively reported living near the clinic. Among these clients, several felt access would be an issue if the time needed to travel to the clinic exceeded an hour. Also, positive and negative ratings alike were linked to concerns about physical and personal obstacles. Physical obstacles included: the challenging terrain (e.g., hills); the lack of good roads and appropriate transport; and the rains. The most common personal obstacle stated was illness (theirs or their child’s), which was mentioned by clients who lived at varying distances from the clinic (3-60 minutes). Other personal obstacles mentioned were: 1) caring for a child on the way to the clinic; 2) having other commitments on the day of the clinic; and 3) husbands being unsupportive of FP.

#### Counselling

Among the clients who rated the counselling positively, several said the provider discussed their reproductive preferences with them. Of these clients, a few mentioned that they would have given a less positive response if the provider had: 1) not demonstrated an interest in their preferences; 2) not asked them about the number of children they wanted; or 3) not provided advice. Similarly, the majority of clients who rated this domain negatively said the provider was not interested in knowing their preferences and did not ask clients about these. However, by far the most common point made by clients who rated this domain positively was that providers allowed them to make their own choices about the number of children they desired and which contraceptive to use.

#### Confidentiality

Positive ratings of confidentiality were linked to the providers’ individual behaviours. For example, clients who gave positive ratings said providers 1) did not disclose their information or choices to others in the clinic, and 2) took steps to facilitate private discussions with clients, such as speaking with lowered voices or isolating FP clients to enhance privacy. Clients also explained their positive ratings by saying that they were counselled individually, which allowed for private interactions with the provider. Conversely, some negative ratings were linked to having received FP services in groups or in pairs. However, most clients who rated this domain negatively explained that the clinic's shelter and use of space undermined confidentiality. They reported receiving FP counselling and contraceptives in an open space where others could overhear or catch sight of their discussions with the FP provider.

#### Environment

In general, clients’ ratings of the clinic’s enironment were linked to their views on the appropriateness of the clinics’ shelter. Specifically, the absence of a shelter, toilet and water were issues mentioned by clients who rated this domain negatively. Additionally, clients who believed the space in the clinic was adequate and clean (e.g. swept or mopped) mostly rated the clinic’s environment positively; whereas, those who believed the space was insufficient gave a negative rating, even if they felt it was clean. However, most clients who perceived the clinic to be unclean rated this domain negatively.

#### Choice of provider

Many clients who rated the choice of provider negatively said the way services were organised in the clinic prevented the opportunity to choose a provider. Clients mentioned needing to respect the clinic’s client flow and the pre-determined roles assigned to providers. Also, perceived staff shortages were believed to hinder the clients’ choice of provider. In contrast, the few clients who rated this domain positively said they could choose the provider that served them in the clinic and that it was their right to do so. Overall, some clients viewed all providers as equals and therefore believed having a choice of provider was not necessary, whilst others felt there were differences among the providers’ capacity that justified the need for a choice.

### Associations between domain ratings and key factors

Associations between the clients’ ratings of the eight responsiveness domains and the following factors were examined: 1) the clinic’s shelter, number of rooms, use of space for FP and immunisations, FP client load, and staffing level; 2) the eight elements of FP counselling presented in Table 2; and 3) the client's age, education, marital status, number of children, travel time to the clinic, exclusive use of one clinic for FP services, and socio-economic status. No significant associations were found between any of these factors and the clients’ ratings of the dignity and environment domains. All significant associations found are presented in Table 4.

**Table 4:** Associations between domain ratings and key factors

Domain	Factor		Positive domain rating		N^a^	***p***-value
			%	n		
**Service continuity**	***Element of FP counselling***					
	Heard the group health talk	Yes	96.7	88	142	0.012
		No	80.4	41		
	Told how different contraceptive methods work	Yes	96.3	77	137	0.022
		No	84.2	48		
	Told about possible contraceptive side effects	Yes	97.3	72	137	0.004
		No	84.1	53		
	Told when to seek follow-up services	Yes	96.7	116	137	<0.001
		No	52.9	9		
	Told where to seek follow-up services	Yes	96.7	109	137	<0.001
		No	66.7	16		
**Communication**	***Element of FP counselling***					
	Heard the group health talk	Yes	94.5	86	141	0.001
		No	78.0	39		
	Asked about past use of contraceptives	Yes	100.0	43	136	0.031
		No	82.8	77		
	Told how different contraceptive methods work	Yes	95.0	76	136	0.004
		No	78.6	44		
	Told about possible contraceptive side effects	Yes	96.0	71	136	<0.001
		No	79.0	49		
	***Client characteristic***					
	Exclusively uses one clinic for FP services	Yes	95.8	46	140	0.021
		No	84.8	78		
**Ease of access**	***Client characteristic***					
	Travelled less than 45 minutes to reach the clinic	Yes	87.0	87	142	<0.001
		No	52.4	22		
**Counselling**	***Element of FP counselling***					
	Asked how many more children are desired	Yes	96.3	26	129	<0.001
		No	56.9	58		
	Asked about past use of contraceptives	Yes	86.1	37	129	<0.001
		No	54.7	47		
	Asked about problems with past contraceptive use	Yes	75.9	44	129	0.028
		No	56.3	40		
	Told about possible contraceptive side effects	Yes	76.4	55	129	0.008
		No	50.9	29		
	***Client characteristic***					
	Exclusively uses one clinic for FP services	Yes	80.4	37	133	0.028
		No	58.6	51		
**Confidentiality**	***Element of FP counselling***					
	Asked about past use of contraceptives	Yes	74.4	32	137	0.020
		No	55.3	52		
	Asked about problems with past contraceptive use	Yes	72.1	44	137	0.009
		No	52.6	40		
	***Client characteristic***					
	Travelled less than 45 minutes to reach the clinic	Yes	67.0	65	139	0.016
		No	50.0	21		
	Exclusively uses one clinic for FP services	Yes	81.3	39	141	0.020
		No	52.7	49		
**Choice of provider**	***Element of FP counselling***					
	Asked how many more children are desired	Yes	48.2	13	136	0.013
		No	22.9	25		
	***Client characteristic***					
	Exclusively uses one clinic for FP services	Yes	40.4	19	140	0.027
		No	22.6	21		

^a^*N* varied from 146 due to missing data

## Discussion

This case study sought to contribute to a deeper understanding of clients’ experiences of FP services that are integrated with childhood immunisations by assessing the responsiveness of these services in terms of eight domains. Overall, the results indicate that in routine outreach clinics, FP services can be responsive when integrated with childhood immunisations, particularly in terms of the dignity and service continuity they afford clients, though less so in terms of the confidentiality, environment, and choice of provider that clients experience. Similar findings were reported by the only other assessment of FP service responsiveness, in which clients in Sri Lanka rated most positively the dignity they experienced and least positively the choice of provider and of contraceptive they were afforded [25]. Despite some lower performing domains, clients interviewed in the case study almost unanimously reported that they would recommend the clinic to a family member or friend interested in FP services. Taken together, these results suggest that the services were likely sufficiently responsive to warrant the repeat use of services, and support the call to integrate FP services with childhood immunisations to reduce the unmet need for contraceptives among postpartum women.

Interestingly, despite being considered most important by clients, the environment and confidentiality were found to be among the responsiveness domains that they rated least postively. This is consistent with findings from the WHO’s general population surveys of health systems responsiveness in which the importance of the environment generally ranked higher among countries with low health expenditure and human development index scores [37]. However, it is possible that the domains considered by clients to be least responsive were also most salient to them, causing clients to rank these among the most important. A notable exception was the choice of provider, which clients’ ranked low both in terms of importance and performance. A possible explanation for this is that clients may not have viewed the choice of provider as a priority given the unmet needs felt in relation to other domains. As De Silva notes, ‘the ability to choose between care providers becomes increasingly important as the other aspects of responsiveness are met’ [18].

Moreover, six of the eight domains’ ratings were found to be significantly associated with several elements of FP counselling and a few client characteristics. Of note, the communication, choice of provider, confidentiality and counselling ratings were positively associated with the exclusive use of one clinic for FP services. This is consistent with findings from Sri Lanka, where clients’ positive responsiveness ratings were associated with using only one FP clinic within the past year [25]. This suggests that experiences with other services may serve as a benchmark and consequently affect clients’ perceptions of the responsiveness of integrated FP services. The influence of a point of reference on clients’ perceptions may also partly explain the unexpected association found in this study between positive ratings of confidentiality and travelling less than 45 minutes to reach the clinic. That is, it is possible that clients who live near a clinic are less likely to seek services elsewhere and thus to have a benchmark, rendering them less critical of the services they experience. Although this is a plausible explanation that is potentially supported by the data, a more conclusive statement cannot be made given the study’s sampling limitations. Further research examining the influence of such a benchmark on perceptions of responsiveness could help improve the delivery of services. Also, contrary to expectations, the clinic’s shelter, number of rooms, and use of space were not significantly associated with clients’ domain ratings, despite results from the cognitive interviewing probes suggesting that infrastructure deficits (e.g., the absence of a suitable shelter) influenced clients’ perceptions of the environment and the confidentiality they experienced. Likewise, counselling ratings were not significantly associated with hearing the group health talk, nor being told how different contraceptives work. It is possible that these two elements of counselling were less relevant to respondents’ perceptions of the counselling domain as the majority of clients interviewed were repeat service users attending the clinics to collect their usual contraceptive method.

The findings also revealed that clients believed that service responsiveness was influenced by the organisation of services in the clinic and the providers’ individual behaviours. Specifically, group-based services were perceived to be less responsive to clients’ needs than one-on-one services in terms of the confidentiality and counselling afforded to clients. Similarly, due to the client flow adopted in the clinics, clients who missed the health talk because they reached the clinic late were deprived from receiving crucial information. Additionally, the pre-determined roles assigned to providers due to the client flow design were believed to prohibit clients’ choice of provider. This echoes recent findings from other empirical studies that demonstrate the important influence of organisational elements on the integrated delivery of FP and childhood immunisation services [9, 10]. It also highlights the value of service designers and implementers adopting a client-centred approach to service organisation in delivery sites. Additionally, the clarity and consistency of the information providers shared with clients, their respect of clients’ choices, the privacy they facilitated, and the professionalism and kindness they exhibited towards clients were found to be central to clients’ experiences. This supports evidence from other studies, including the Integra initiative, which emphasise the crucial role played by providers in the delivery of integrated services and how their individual performance largely determines the success of integration programmes [11, 38–42].

Furthermore, this study corroborates previous research that established the value of combining cognitive interviewing techniques with Likert questions to test whether questions fulfil their intended purpose [33, 34, 36, 43, 44]; and provides an example of its applicability to the assessment of service responsiveness. The cognitive interviewing probes helped to qualitatively validate the tool used in this study and the clients’ responsiveness scores by confirming that the Likert questions and scale were well understood and accurately interpreted by respondents. The data derived from these probes also yielded important insights into clients’ experiences that would not have otherwise been captured. Using a similar method, Scott *et al*. demonstrated that Likert questions and scales were not well suited to capturing respondents’ experiences of respectful maternity care in rural northern India [45]. Specifically, they found that Likert response options were often misunderstood, hypothetical questions were commonly misinterpreted, and the translation of standard terms from the literature did not resonate well with respondents in the studied context. It is possible that the successful use of Likert questions in this case study resulted from the approach adopted to refine and pilot the data collection instrument, which was heavily informed by experienced local interviewers. Futher research is needed to examine the applicability of this combination of methods to the assessment of service responsiveness in different contexts.

## Limitations

This study has a few limitations worth noting. First, the exit interviews were susceptible to response biases, such as courtesy and desirability biases. However, the clients’ responses to the cognitive interviewing probes suggest that these biases were likely minimal. Second, in some cases, the providers’ prior knowledge of the researchers’ visit and the presence of the data collection team in the clinics may have prompted providers to alter their delivery of services, rendering them more or less responsive than usual. Third, sub-group analyses and the inferences that could be derived from the quantitative data were restricted by the small sample of clients interviewed. Nonetheless, the findings from the cognitive interviewing probes offer important insights that help mitigate this limitation. And finally, since the study’s primary aim was to assess clients’ experiences of FP services that were integrated with childhood immunisations, comprehensive data were not captured on the growth monitoring services delivered in the studied clinics. This represents an important limitation as the results show that the majority of clients had the intention to seek both growth monitoring and FP services on the day of the exit interviews, which was likely due to the more frequent need for these services compared to scheduled immunisations.

## Conclusions

This case study set out to investigate clients’ experiences of FP services that were integrated with childhood immunisations in routine outreach clinics. In doing so, it also demonstrated the value of combining cognitive interviewing techniques with Likert questions to assess service responsiveness. The results from this study establish that in routine outreach clinics, FP services can be responsive when integrated with childhood immunisations, particularly in terms of the dignity and service continuity they afford clients, though to a lesser extent in terms of the confidentiality, environment and choice of provider experienced. The clients’ views of the choice of provider, communication, confidentiality and counselling they experienced were found to be positively associated with the exclusive use of one clinic for FP services, suggesting that having a benchmark may have an important influence on perceptions of responsiveness. The findings also highlight the influence of the organisation of services and of the providers’ individual behaviours on service responsiveness. Further research is therefore needed to interrogate the views of providers and their influence on the responsiveness of FP services that are integrated with childhood immunisations.

## Data Availability

The datasets used and/or analysed in the current study, as well as the data collection instruments, are available from the corresponding author on reasonable request.

## Abbreviations

EPI: Expanded Programme on Immunisation
FP: Family planning
HSA: Health surveillance assistant
